# A Review on Microcellular Injection Moulding

**DOI:** 10.3390/ma14154209

**Published:** 2021-07-28

**Authors:** Yifei Ding, Mohammed H. Hassan, Otto Bakker, Srichand Hinduja, Paulo Bártolo

**Affiliations:** Department of Mechanical, Aerospace and Civil Engineering, The University of Manchester, Manchester M13 9PL, UK; yifei.ding@manchester.ac.uk (Y.D.); Mohamed.hassan@manchester.ac.uk (M.H.H.); ottojan.bakker@manchester.ac.uk (O.B.); sri.hinduja@manchester.ac.uk (S.H.)

**Keywords:** mechanical properties, microcellular injection moulding, MuCell^®^, polymer processing, processing parameters, surface quality

## Abstract

Microcellular injection moulding (MuCell^®^) is a polymer processing technology that uses a supercritical fluid inert gas, CO_2_ or N_2_, to produce light-weight products. Due to environmental pressures and the requirement of light-weight parts with good mechanical properties, this technology recently gained significant attention. However, poor surface appearance and limited mechanical properties still prevent the wide applications of this technique. This paper reviews the microcellular injection moulding process, main characteristics of the process, bubble nucleation and growth, and major recent developments in the field. Strategies to improve both the surface quality and mechanical properties are discussed in detail as well as the relationships between processing parameters, morphology, and surface and mechanical properties. Modelling approaches to simulate microcellular injection moulding and the mathematical models behind Moldex 3D and Moldflow, the two most commonly used software tools by industry and academia, are reviewed, and the main limitations are highlighted. Finally, future research perspectives to further develop this technology are also discussed.

## 1. Introduction

Plastics, due to their excellent mechanical properties, low density, and corrosion resistance, are widely used in different fields such as aerospace and automotive, packaging, building construction, and healthcare [1,2]. In 2019, the global production of plastic materials reached 368 million metric tons, with the European production representing around 16%, and it is forecasted that plastic production will increase to 1.12 billion tons by 2050 [3].

Most plastics are fossil-based, and there are significant concerns regarding the environmental impact of their use. However, researchers are making significant progress regarding the development of bio-based polymers that represent around 1% of the total market [4,5]. Plastic parts can be produced through a wide range of techniques, such as injection moulding, compression moulding, extrusion, blow-moulding, thermoforming, and reaction-injection moulding [6,7,8,9]. Among these technologies, injection moulding is the most relevant technique.

An injection moulding system consists of an injection unit, a mould closing unit, an ejection unit, a core pulling unit, and a cooling unit. The main target of the injection unit is to melt the plastic material and inject it into the mould cavity. The main injection unit components are the screw inside a screw chamber, heating elements around the screw chamber, and a hopper that contains the raw material. The screw, heating elements, and screw chamber act together. They melt the plastic material, decreasing its viscosity and increasing its flowability. The screw moves forward inside the screw chamber and pushes the molten polymer into the mould cavity, which increases density and decreases shrinkage. Therefore, the injection moulding cycle can be summarised as follows [10,11,12]:

- Plastic injection;
- Holding and packing;
- Cooling and solidification;
- Mould opening and part ejection.

**Figure 1:**
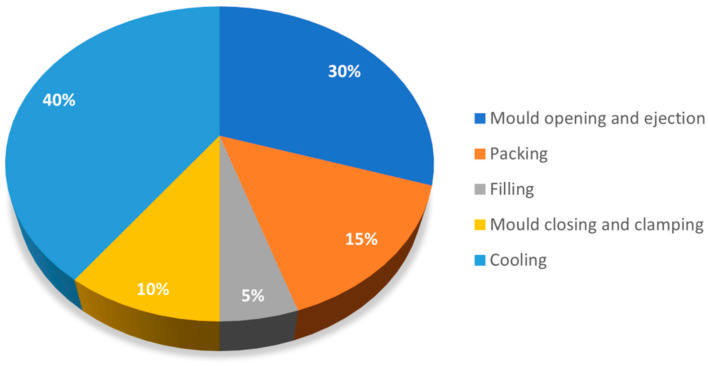
The cycle time of injection moulding (figure adapted from [13]).

Figure 1 shows the average percentage of each phase over the overall injection moulding cycle [13]. The total cycle depends on different factors, of which the part wall thickness is one of the most relevant. Nevertheless, the cooling stage is always the more time-consuming step, representing more than half of the injection moulding cycle.

An important market for injection moulding parts is the automotive sector. In the European Union (EU), this is a sector under significant safety and environmental regulations. Restrictions on CO_2_ emissions imposed by the EU led not only to the development of new-energy powered vehicles, such as hybrid and electric vehicles, but also to the development of more efficient and light-weight gasoline-powered vehicles. Therefore, the automotive industry is increasingly demanding high-performance and light-weight plastic parts. Thus, injection moulding companies supplying plastic parts for the automotive sector are facing significant challenges, as current injection moulded parts must be redesigned, and new injection moulding strategies are required.

Replacing solid injected moulded parts by foamed ones represents an effective way to reduce part weight [14,15,16]. Thermoplastic foaming parts can be produced using two types of blowing agents: chemical and physical blowing agents [15,17,18]. In the case of chemical blowing agents, the agents are mixed with the polymeric materials in the hopper and moved into the barrel. When the temperature reaches a certain value, gas such as nitrogen, carbon dioxide, or carbon monoxide is released, creating an internal microcellular structure [19,20]. The main disadvantages of using chemical blowing agents are related to uneven bubble formation and difficulties in dealing with the remaining chemical by-products in the machine [20].

The microcellular injection moulding is a foaming technology that uses a physical blowing agent. MuCell^®^ was the first commercialised microcellular injection moulding process being also the most known technique [16,21]. However, other technologies were recently developed and commercialised such as Optifoam^®^, ProFoam^®^, Ergocell^®^ [16,21,22], and IQ Foam^®^ [23]. All of these technologies are based on the mixture of a gas/supercritical fluid (SCF) and the melt during the injection moulding process, but involving different mixture methods [16,21,22,23]. In the MuCell^®^ process, a specially designed reciprocating screw is used as the SCF dosage element. This screw, longer than a conventional one, is equipped with a mixing section designed to optimise the SCF-polymer melt. The Optifoam^®^ process uses a specially designed nozzle as the SCF dosage equipment. In the ProFoam^®^ process, the gas is put into the hopper straight and dissolves with the melt inside the injection unit, while in the Ergocell^®^ process, a dynamic mixer is used for mixing SCF with the melt. Finally, in the IQ Foam^®^ process, a two-chambered unit is set up between the hopper and the screw chamber to make the melt and gas mix at moderate-low pressures [16,21,22,23]. Among these technologies, MuCell^®^ has the highest industrial acceptance and is the leading technology. These technologies, and MuCell^®^ in particular, allow not only to produce light-weight plastic parts but also to reduce carbon footprint and CO_2_ emissions [24]. 

This paper provides an overview on recent advances in the MuCell^®^ technology, discussing in detail strategies to address critical problems related to surface quality and mechanical properties of produced foamed parts. As numerical simulation is a standard task of companies producing injection moulding plastic parts, the use of commercially available software tools is discussed, and the considered mathematical models are reviewed. Finally, we highlight key challenges still to overcome within the field.

## 2. The MuCell^®^ Process

The MuCell^®^ microcellular injection moulding process was invented at MIT (Massachusetts Institute of Technology, Cambridge, MA, USA), aiming at reducing the weight and production costs of plastic parts [25]. This process allows 30% to 40% material reduction and yields parts with higher impact strength and an internal structure consisting of a large density of small bubbles (2 to 10 µm) [25,26,27]. The viscosity of the polymer melt in the barrel is also reduced due to the mixing with a supercritical fluid [28,29]. Initially, the microcellular foaming was achieved by batch process, and as a consequence, the cycle time was very long, and the size of foamed bubbles is very large. Later, Trexel (Wilmingon, MA, USA) improved this technology by integrating it with an injection moulding machine through a continuous process commercialised under the name of MuCell^®^ Moulding [30].

**Figure 2:**
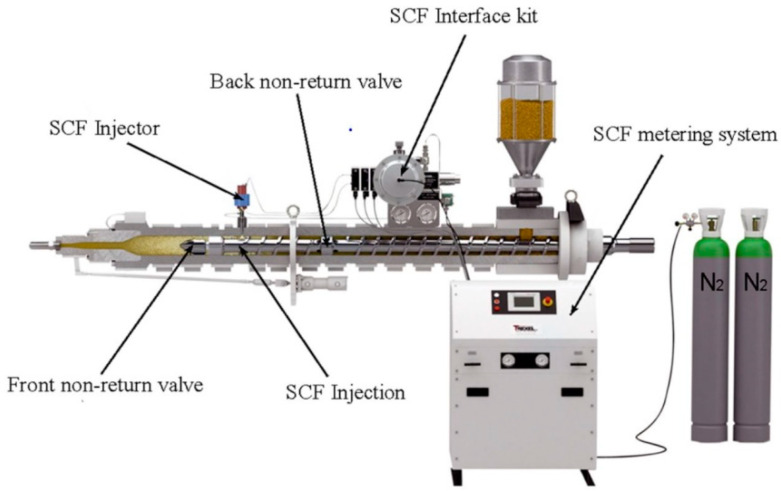
The structure of a typical MuCell^®^ system [23].

**Figure 3:**
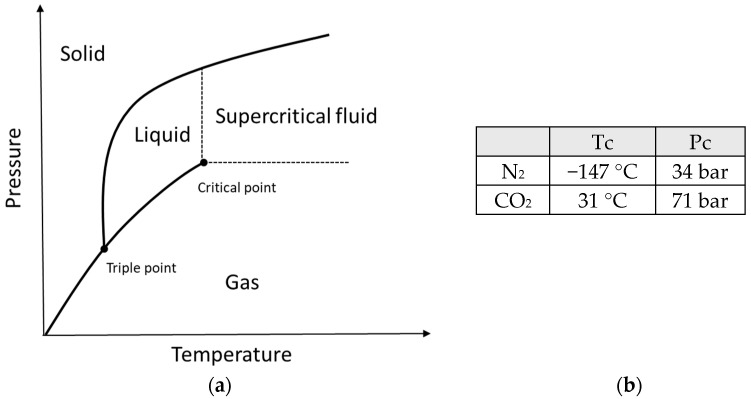
(**a**) Definition of a supercritical fluid status, (**b**) Critical temperature (Tc) and critical pressure (Pc) of N_2_ and CO_2_.

The structure of a typical MuCell^®^ machine is shown in Figure 2. It consists of an inert gas pump, SCF metering system, SCF injector, front and back non-return valves, and shut-off nozzle. In the SCF metering system, there is a mass flow element controlling the SCF level mixed with the molten polymer [31,32]. The shut-off nozzle is used for preventing the melt flowing back through the nozzle [30,33]. The main MuCell^®^ process can be summarised into four main steps [21,33,34,35,36]: SCF mixing and dissolution in the polymer melt, cell nucleation, cell growth, and solidification. In the gas dissolution step, inert gas is pressurised to a SCF state and then goes through the mass flow element to be mixed with the melted polymer inside the screw chamber between the front and back non-return valves. The SCF phase is achieved by injecting the gas above its critical pressure (Pc) and critical temperature (Tc) (Figure 3). Mixing the SCF under pressure enhances the solubility within the polymer melt [37]. The most commonly used inert gases are carbon dioxide, which presents high solubility, and nitrogen, which allows a higher foaming level [20]. Other gases such as argon and helium were also investigated, but they are more expensive and flammable and induce machine degradation [16]. Cell nucleation starts when the mixture of SCF and the melt is injected into the mould cavity, and this process is caused by a rapid pressure drop [20,21,23,31,38,39,40]. Cells keep growing and enlarging, while the mixture continues being injected into the mould cavity and the gas–polymer melt remains at an elevated temperature. In the last step, the growth of the cells is stopped by the cooling effect, and the solidified plastic part is ejected from the mould cavity. 

Cell nucleation comprises two main mechanisms: homogeneous and heterogeneous nucleation [27,40]. Homogeneous nucleation occurs when the gas is dissolved into a homogeneous polymer melt, without any impurities or additives. Heterogeneous nucleation occurs when bubbles form at two different phases such as the polymer and an additive. In this case, the nucleation occurs on the surface between the additives or filers and SCF–polymer melt. Generally, the heterogeneous nucleation rate is faster than the homogeneous nucleation due to the lower activation energy [40,41,42,43,44,45]. Usually, polymers are mixed with a wide range of additives that do not allow creating a homogeneous mixture, thus resulting in a small number of bubbles but large bubbles. In order to produce a large number of small bubbles, additives must be added to the polymer [43,44,45].

Moon et al. [46], using polypropylene (PP), correlated the bubble size with the mechanical properties of microcellular parts, observing that smaller bubble sizes allow for better mechanical properties. They also found that the increase in the gas saturation pressure increases the bubbles density and decreases both the energy barrier for nucleating stable bubbles and bubbles diameter. The results also showed that the gas saturation pressure limited the bubble growth in very short times. Despite the good agreement between experimental work and theory, differences were also reported due to simplifications of the theoretical model.

Dong et al. [47], using acrylonitrile butadiene styrene (ABS), investigated the cell structure of microcellular injection-moulded parts along the vertical and parallel direction to melt flow and found that both round and distorted cells were present in the moulded part. By analysing the cell foaming mechanism of MuCell^®^, the authors concluded that those two types of cell shapes were formed at different stages. The distorted cells were formed in the filling stage, and the shape was contributed by the fountain-flow effect, while the round cells were formed in the cooling stage due to the cooling shrinkage. The results showed that during the MuCell^®^ process, the cell formation occurred in two phases (filling and cooling) due to melt pressure differences.

Colton et al. [41] investigated the microcellular foaming process on semi-crystalline polypropylene and the effect of different additives on the foaming process. Contrary to what was observed for amorphous materials [43,44,48], semi-crystalline polymers contain very long chains in a uniformly arranged order. Therefore, the processing temperature is higher, and the presence of compact crystalline areas limits the space for dissolving the gas. Moreover, crystallites could also physically stop the foaming process. However, the authors found that the nucleation mechanism of semi-crystalline polymers is similar to the amorphous polymers. Behravesh and Rajabpour [32,49] studied the cell formation in the filling stage using high-impact polystyrene and found that the shot size was the dominant factor influencing both cell formation and growth. As observed, if the shot size was less than 80%, the mould cavity was not fully filled, but the foam percentage in the produced parts was higher. If the shot size was greater than 80%, the mould cavity was fully filled, but the produced parts exhibit low foaming values or even no foaming. The authors also verified that in the case of less cell formation, the gas was still dissolved in the polymer, as there is not enough time for cell nucleation and growth.

Several authors [38,50,51] also demonstrated that the core-back process facilitates the nucleation process due to the rapid pressure drop achieved by retracting the moving part of the mould after the cavity was filled and a time delay to allow the solid skin to be formed. This allows for high cell fractions and a higher reduction of the weight and stiffness-to-weight ratio.

## 3. Silver Marks and Solid Skin Formation

**Figure 4:**
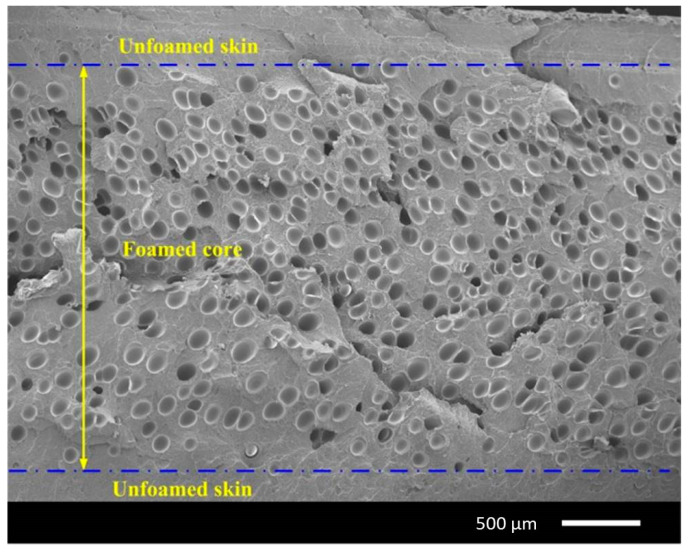
The typical cross-section structure of a microcellular injection moulded part observed using scanning electron scope (SEM) [47].

**Figure 5:**
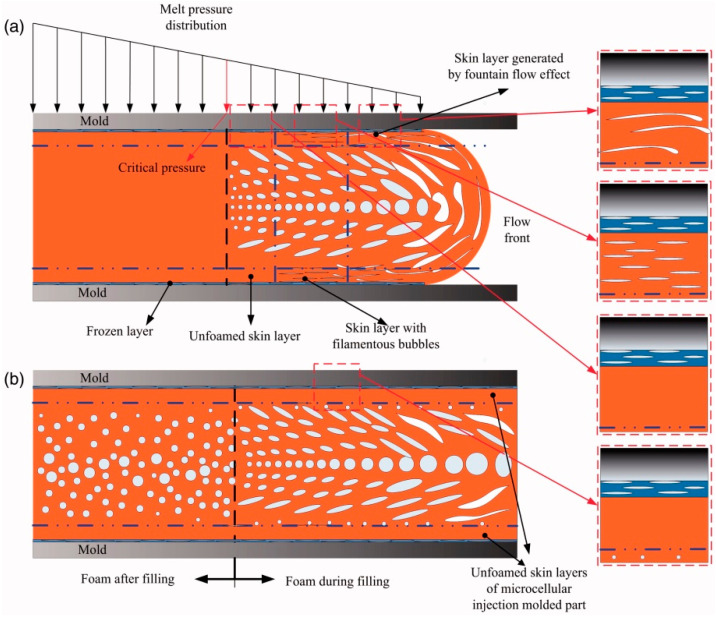
Foaming process and solid skin formation during the filling stage (**a**) and the cooling stage (**b**) [56].

Typically, a microcellular injection moulded part presents a sandwich cross-section structure of a microcellular injection moulded part consisting of a solid skin and foamed core (Figure 4). Lee and Cha [52] found that the formation of a solid skin layer is mainly due to a low mould temperature that prevents the cell growth in the molten plastic. Moreover, the skin layer thickness can be influenced by both the flow rate and the mould cavity depth [52,53,54,55]. According to Wang et al. [21], the solid skin formation occurs at both the filling and the cooling stages. Two main effects contribute to the solid skin formation during the filling stage. The first effect is the re-dissolution of the gas in the skin layer. The second effect corresponds to the high cavity pressure at the injection gate, which compromises the cell formation process. During the cooling stage, cells can be formed in regions close to the mould walls, which decrease the thickness of the solid skin layer. Dong et al. [56] also found that the solid skin layer formation occurs at both the filling and cooling stages. However, according to these authors, the solid skin formation is mainly due to the shear flow and fountain flow during the filling phase and the cooling and polymer solidification process (fast cooling near the mould walls) during the cooling stage. Figure 5 details the solid skin formation during both the filling and cooling stages.

**Figure 6:**
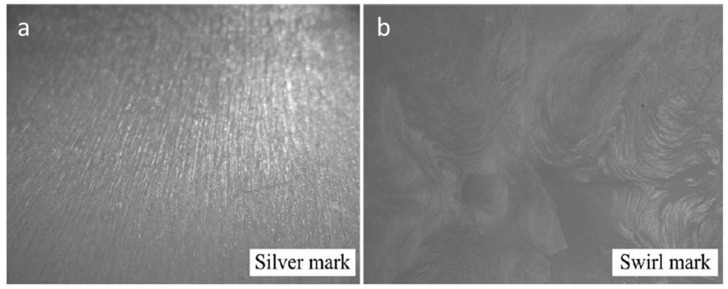
Silver marks on products surface made by microcellular injection moulding, (**a**) At the low injection velocity region, (**b**) At the high injection velocity region) [21].

Silver marks are one of the two major defects of microcellular injection moulding. It usually shows up on the microcellular injection moulded product surface, as shown in Figure 6. This defect prevents MuCell^®^ wide applications on areas requiring good surface quality, such as electronic items and control panels [57]. The mechanism of silver mark formation is associated to the use of a mould cavity temperature much lower than the glass transition temperatures or crystalline temperature, so that the gas is trapped between the cavity wall and cooled molten plastic and cannot dissolve into the molten plastic again [21,57].

There are two methods to remove silver marks from the part surface [58,59,60]. One method consists of preventing cell foaming in the flow front during the filling stage, while the second method consists of making trapped gas between the melt and cavity wall dissolve into the melt [21]. 

**Figure 7:**
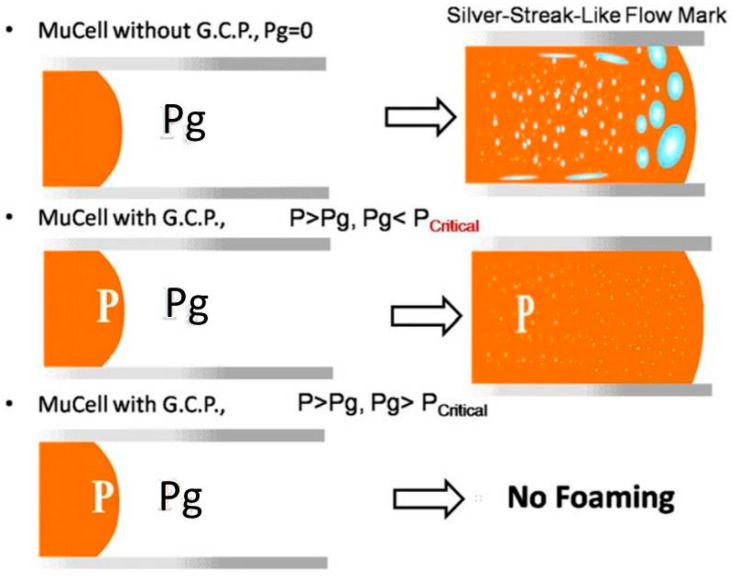
The influences of GCP on cell formation [62].

**Figure 8:**
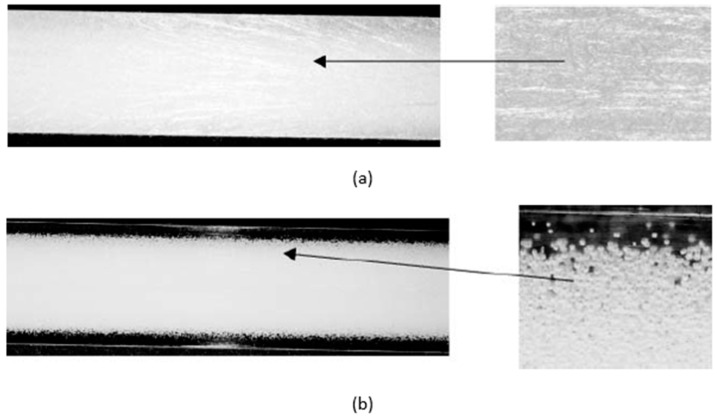
Surface of a test bar made by MuCell^®^ (**a**) and MuCell^®^ with gas counter pressure (**b**) [61].

For the first method, a gas counter pressure (GCP) system was developed [22,23]. This technology (Figure 7) combines mould pressure control equipment with the MuCell^®^ system. By increasing the mould cavity pressure to a certain level in the filling stage, using the mould pressure control equipment, cell foaming is significantly restricted, and cells are small and round. Figure 8 shows the surface appearance of test samples produced using MuCell^®^ and MuCell^®^ with GCP considering the same weight reduction. Results showed that the surface roughness of the sample made by MuCell^®^ and GCP was significantly lower (0.85 µm (Rz)) than the surface roughness of parts produced by MuCell^®^ without GCP (23.11 µm (Rz)) [61].

**Figure 9:**
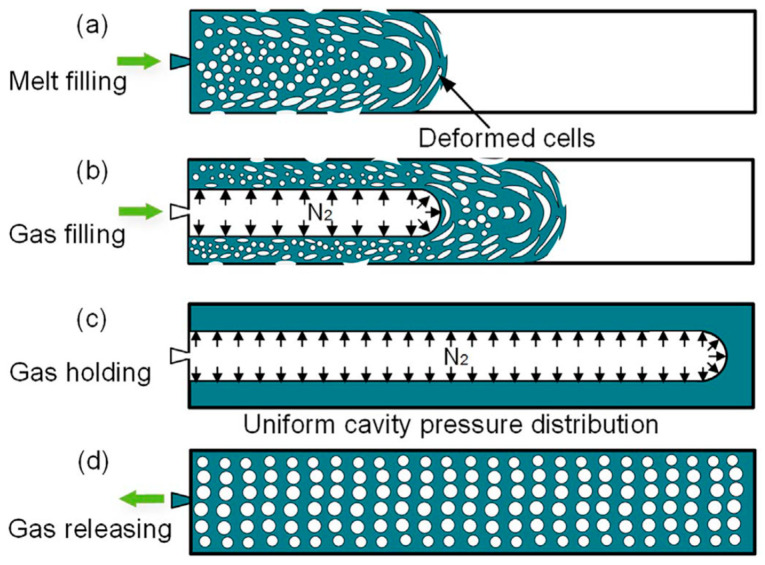
The principle of GAMIM, (**a**) Melt filling stage, (**b**) Gas filling stage, (**c**) Gas holding stage, (**d**) Gas releasing stage [63].

**Figure 10:**
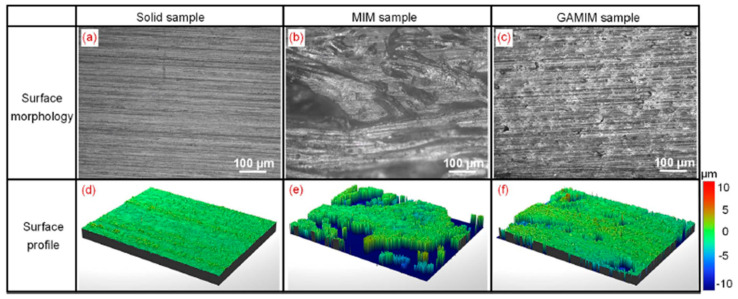
Surface morphology of parts produced by conventional injection moulding, microcellular injection moulding (MIM) and GAMIM processes (**a**) Surface morphology of the Solid sample, (**b**) Surface morphology of the MIM sample, (**c**) Surface morphology of the GAMIM sample, (**d**) Surface profile of the Solid sample, (**e**) Surface profile of the MIM sample, (**f**) Surface profile of the GAMIM sample [63].

Hou et al. [63] developed the gas-assisted microcellular injection moulding (GAMIM) method to improve the surface quality of products produced by MuCell^®^. This method, as illustrated in Figure 9, combines gas-assisted injection moulding (GAIM) and microcellular injection moulding (MIM). In the filling stage, the pressurised gas from the GAIM system is injected into the molten plastic to fully fill the mould cavity, as shown in Figure 9a,b. After the filling stage, the injected gas is held for a certain time to maintain the mould cavity under high pressure (Figure 9c). After the gas released from the mould cavity, the cell foaming process occurs due to a pressure drop (Figure 9d). Figure 10 shows the surface quality of solid, MIM, and GAMIM samples. As observed, the surface quality is higher in the GAMIM-injected sample, as the high-cavity pressure makes all the formed cells re-dissolve into the plastic melt at the gas holding stage.

**Figure 11:**
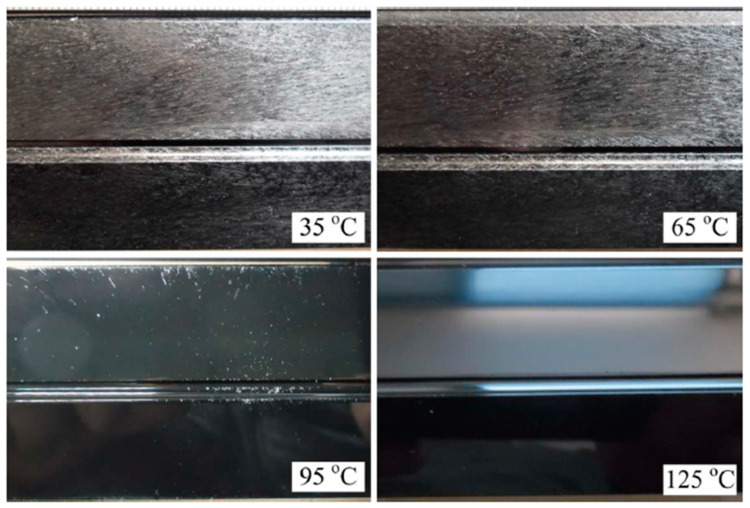
Surface appearance of MuCell^®^ parts under different mould temperature during the filling stage [21].

Regarding the second method, Wang et al. [21] applied a rapid mould heating and cooling (RMHC) system in the MuCell^®^ process, allowing quickly controlling the mould heating and cooling process. The RMHC enables increasing the mould temperature to values higher than the material glass transition temperature during the filling stage and rapidly decreasing the mould temperature during the cooling stage [64]. Moreover, the authors found that high mould temperatures in the filling stage do not only eliminate silver marks but also enhance the surface gloss and reduces surface roughness, as shown in Figure 11. This is because the high mould cavity temperature during the filling stage leads the trapped gas on the part surface to dissolve into the melt again.

Dong et al. [65] obtained similar results by increasing the mould temperature in the filling stage of MuCell^®^ using a dynamic mould temperature control system. This dynamic mould temperature control system uses electric rod and cooling water for heating and cooling the mould. As observed, under a high mould temperature, most of the gas trapped between the cavity wall and the melt surface escapes to the air, and only a small amount dissolves into the high-temperature melt, which explains the observed elimination of silver marks.

**Figure 12:**
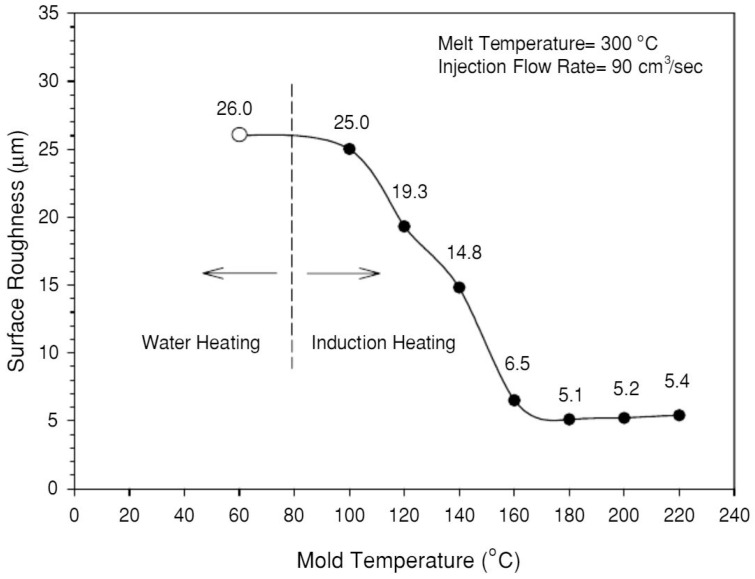
The trend between mould temperature and surface roughness of microcellular injection moulded parts [66].

Chen et al. [66] applied an electromagnetic induction heating technology and a water cooling system to rapidly change the mould temperature in the filling stage. Experimental tests were conducted using polycarbonate (PC), and the results showed that the silver marks can be completely removed when the mould temperature is higher than 160 °C. The surface roughness also decreases by increasing the mould temperature up to 180 °C, after which the surface roughness increases, as shown in Figure 12. However, this study requires further developments, as no explanation is provided regarding the increase of the surface roughness for temperatures between 180 °C and 220 °C. 

Chen et al. [59] developed a novel pressure-temperature (P-T) control system by combining a GCP and mould temperature control system together in the microcellular injection moulding process, obtaining parts with small and uniform cells and no silver marks. Although the combined use of GCP and a mould temperature control system can successfully eliminate silver marks, the use of GCP alone increases the solid skin thickness, which compromises the part weight reduction, while the individual use of a mould temperature control system increases the size of the cells and contributes to an uneven distribution of cells in the MuCell^®^ parts. Furthermore, it was found that the critical parameters in the P-T control system, such as gas pressure holding time, relief time, and the mould cooling speed result in different cell morphologies [67]. Longer relief times and faster cooling speeds, which can be induced by controlling the holding time, originated thick skin thicknesses and small cell sizes. 

Yoon et al. [68] investigated the effect of mould surface coating to improve the surface quality of microcellular injection moulded parts. In this case, the mould cavity was coated with a PEEK layer through a thermal spray method to slow down the heat transfer process during the microcellular injection moulding process. Two materials were considered in this research: PP and ABS/PC. The results showed that this strategy allowed obtaining MuCell^®^ parts with surface quality as good as solid injected parts for both materials. Similar results were obtained by Chen et al. [69] that coated the mould cavity using Bayfol polycarbonate/polyethylene terephthalate polyester film (82% PET + 18% PC) through an In-Mold Decoration approach. PC was the material used in this research. The authors also investigated the effect of the coating layer thickness (increased from 0.125 to 0.188 mm), observing that silver marks decrease by increasing the layer thickness (no silver marks at 0.188 mm).

All silver-marks-removal methods mentioned above need additional equipment or treatments in MuCell^®^ process; this will increase the manufacture cost. Lee et al. [70] came up with an novel idea to remove silver marks on MuCell^®^ products surface without introducing any additional equipment. The idea is to control the cell nucleation of polymer/gas solution by changing the material formulation and gas concentration. They found that lowering the gas content under a certain value can make the surface roughness of microcellular injection moulded parts comparable to that of conventional injection moulded parts. The reason is that low gas content contributes to the high activation energy of cell nucleation and low cell nucleation rate; thus, the cell nucleation and formation are delayed on the melt flow front in the filling stage of MuCell^®^.

Gómez et al. [34] investigated the influence of different injection moulding parameters on the surface roughness of MuCell^®^ products. After analysing a series of experiments, they found that the surface quality of MuCell^®^ parts can be improved significantly by increasing both mould temperature and injection velocity. Low mould temperature, slow injection velocity, little shot volume, and much gas content contribute to poor surface quality.

As reported, different research groups have proposed alternative methods to minimise or even remove the silver marks from the parts surface, but these methods are still for laboratory applications and not yet applied for mass production. Some methods such as the GCP control, mould temperature control, and gas-assisted injection moulding require additional equipment that increases the manufacturing costs. These systems correspond to low-cost options compared to conventional MuCell^®^, but still, they are significantly more expensive than conventional injection moulding. Moreover, GCP and gas-assisted injection moulding significantly increases the cycle time, which has an impact on the final costs. Similarly, core-back injection moulding requires a specially and costly designed mould. Furthermore, there are no systematic studies on how microcellular injection moulding parameters influence surface defects. Alternatively, some researchers explored the use of new additives producing nano-size cells during the injection process.

## 4. Methods for Improving Mechanical Properties

Poor mechanical properties represent another limitation of microcellular injection moulded products. It is found that the mechanical properties of MuCell^®^ parts are related to the inner morphology, such as skin thickness, cell size, and density [62]. Therefore, the relationships between process parameter, cell morphology, and mechanical properties need to be fully understood to allow MuCell^®^ products with good mechanical properties.

Some methods developed for silver-mark elimination can also improve the mechanical properties. It has been shown that the GCP technology can improve the tensile strength of MuCell^®^ products by promoting thicker surface layers [61,62]. Moreover, the combined use of GCP with a mould temperature control system allows improving both the tensile and impact strength [62]. Products made using the GAMIM method also exhibit good mechanical performance on the tensile test, flexural test, and impact test due to its improved inner structure with smaller and more uniform cells and a compact solid skin layer [63].

Gómez et al. [34] investigated the influence of injection moulding parameters including shot volume, mould temperature, and injection velocity on the mechanical properties of MuCell^®^ parts. Results showed that the most influential parameter is the shot volume followed by the mould temperature and the injection velocity. As observed, by increasing the shot volume, the elastic modulus and yield strength also increases. However, the effects of both mould temperature and injection rate on tensile strength and elastic modulus are not significant.

By investigating the correlation between process parameters, cell morphology, and mechanical properties, Bledzki et al. [71] pointed out that both bending and tensile results were influenced by the process parameters in the same way. Higher injection velocity can improve both bending and tensile properties. Furthermore, higher melt temperature and mould temperature cause poor bending and tensile performance of MuCell^®^ products.

As for impact strength, researchers found that it could be impacted by changing both mould temperature and injection velocity [56,72]. Effectively, impact strength seems to decrease by increasing the mould temperature and injection due to the formation of a thinner solid skin layer. However, it was observed that the melt temperature has little effects on the solid skin thickness [56]. Kastner et al. [73] investigated the biaxial bending and flexural behaviour of foamed parts after changing seven process parameters, including melt/mould temperature, degree of foaming, injection speed, delay time, gas content, and back pressure. They found that the mould temperature, degree of foaming, and delay time had the greatest influence on both the bending and flexural properties of foamed parts. In contrast, the influence of melt temperature, gas content, and injection pressure on the mechanical properties is negligible. Moreover, increasing the solid skin thickness increases biaxial bending and flexural performance. 

Gómez et al. [74] investigated the effect of glass fibres on the mechanical properties of MuCell^®^ parts and found an improvement of both flexural and impact properties in areas where the fibres are oriented in a preferential direction. Fracture toughness is also enhanced, as the fibres located in the perpendicular direction prevent crack propagation.

Wang et al. [5] carried out a study on the effect of talc on the mechanical properties of microcellular injection moulded parts. As observed, PP/talc micro-composite foamed parts exhibit better tensile strength performance than PP foamed parts but worse ductility performance. The PP-talc nanocomposite foamed parts also present better strength and toughness than PP counterpart foams.

Sun et al. [75] investigated different polymer blends aiming to improve the ductility and toughness of foamed parts. Polypropylene/high-density polyethylene (PP/HDPE), polypropylene/low-density polyethylene (PP/LDPE), and poly (lactic acid)/poly (3-hydroxybutyrate-co-3-hydroxy-valerate) (PLA/PHBV) blends were investigated considering different weight ratios of the two materials in each blend. They found that both ductility and toughness were significantly higher in PP/HDPE foamed parts with a PP/HDPE weight ratio of 75/25, and PP/LDPE and PLA/HDPV foamed parts weight ratio of 75/25 and 70/30, respectively.

Lee et al. [76] investigated the effect of adding nanoclays and PP on the tensile properties of LDPE foamed parts. As observed, the addition of PP or nanoclays significantly improved the tensile properties, as smaller and denser bubbles were produced in comparison to microcellular LDPE foams without these additives.

Yan et al. [77] used in-mould decoration and MuCell^®^ to produce PP/nano-CaCO_3_ foamed parts showing that the addition of nano-CaCO_3_ (ranging from 0 to 10 wt%) improved mechanical properties and acted as a heterogeneous nucleating agent. The authors also found that the surface quality first decreases with the increase of nano-CaCO_3_ and then increases. As observed, high contents of nano-CaCO_3_ promote the agglomeration of the nanoparticles, which leads to a reduction of the nucleation points. Moreover, a high level of nano-CaCO_3_ also reduces the melt flow rate and increases the melt strength. Optimal results of both surface quality and mechanical properties were achieved in foamed parts containing 6 wt % of nano-CaCO_3_. Similar results were also observed by Llewelyn et al. [78] and Ding et al. [79]. 

Gómez-Monterde et al. [80] investigated the relationship between cell morphology and tensile properties of microcellular injection moulded cylindrical bars under 10% and 17% weight reductions. They found that there were no significant changes on cell morphology including cell size, cell density, and solid skin between the two weight reduction bars. Results showed that elastic modulus and yield strength decreased with the weight reduction. However, both solid and foamed parts exhibited similar yield strain and ultimate strength.

Tao et al. [81] investigated the hybridisation effect of polypropylene-based compounds, which includes hollow glass bubbles (HGBs) and jute fibre, on mechanical properties of MuCell^®^ parts. From tensile tests results, MuCell^®^ moulded parts showed reductions in tensile strength (up to 42%), tensile modulus (up to 56%), yield strain (up to 10%), and breaking strain (up to 9%) in comparison to their solid counterparts, especially for the compounds with a higher level of fillers. These reductions are caused by the non-uniform porous structure inside the MuCell^®^ parts, and a high percentage of fillers will contribute to cell collapse and coalescence. As a consequence, the impact strength of MuCell^®^ moulded parts also decreased. However, the decrease of mechanical properties is still within the acceptable range for automotive component. As for flexural test results, there was no significant changes between MuCell^®^ moulded parts and conventional injection moulded parts. This is because flexural properties mainly depend on the solid skin thickness [82].

## 5. Simulation of the MuCell^®^ Process

Moldflow (Autodesk, San Rafael, CA, USA) and Moldex 3D (CoreTech System Co., Ltd., Chupei, Taiwan) are the two most commonly used commercial software for simulating the MuCell^®^ process. In both cases, the flow field can be simplified by considering mass, momentum, and energy balance equations, as follows [83,84]:

(1) $$\frac{\partial \rho }{\partial t}+\nabla \cdot \rho u=0$$

(2) $$\frac{\partial }{\partial t}\left(\rho u\right)+\nabla \cdot \left(\rho uu+pI-ɳ\left(\nabla u+\nabla u^{T}\right)\right)=\rho g$$

(3) $$\rho C_{P}\left(\frac{\partial T}{\partial t}+u\cdot \nabla T\right)=\nabla \left(k\nabla T\right)+ɳ{\dot{\gamma }}^{2}$$

where ρ is the density of the polymer, *t* is the injection time, u is the velocity vector, ɳ is the viscosity, p is the pressure, *T* is the temperature, $C_{P}$ is the specific heat, I is the unit tensor, g is the gravity, k is the thermal conductivity tensor, and $\dot{\gamma }$ is the shear rate.

In the case of bubble nucleation and growth process, the 3D numerical simulation is applied to describe the dynamic behaviour of the bubble growth, which is coupled with macroscopic molten polymer flow. In both software tools, the radius of bubble growth is given as [85,86]:

(4) $$\frac{dR}{dt}=\frac{R}{4ɳ}\left(P_{D}-P_{C}-\frac{2\gamma }{R}\right)$$

where *R* is the bubble radius, $P_{D}$ is the bubble pressure, $P_{C}$ is the ambient pressure, and γ is the surface tension.

In the case of Moldex 3D, a thin boundary layer condition is assumed, and the dissolved gas concentration profile along the radial direction of a thin shell is described by a diffusion equation as shown below [85]:

(5) $$\frac{\partial c}{\partial t}=D\left[\frac{1}{r^{2}}\frac{\partial }{\partial r}\left(r^{2}\frac{\partial c}{\partial r}\right)\right]$$

where c is the dissolved gas concentration, r is the distance from the center of the bubble, and D is the diffusion coefficient.

In the Moldex 3D, the dynamic bubble growth behavior is also described by the mass transfer at the interface of the gas bubble and as previously proposed by Han and Yoo [87]:

(6) $$\frac{d\left(P_{D}R^{3}\right)}{dt}=\frac{6D\left(R_{g}T\right)\left(c_{\infty }-c_{R}\right)R}{-1+{\left\{1+\frac{2/R^{3}}{R_{g}T}\left(\frac{P_{D}R^{3}-P_{D0}R_{0}^{3}}{c_{\infty }-c_{R}}\right)\right\}}^{1/2}}$$

where the concentration of gas follows the following equation,

(7) $$\frac{c_{\infty }-c}{c_{\infty }-c_{R}}={\left(1-\frac{r-R}{\delta }\right)}^{2}$$

where $c_{\infty }$ is the concentration of the gas dissolved in the melt far from the bubble, which may be considered to remain constant during the entire period of bubble growth, $P_{D0}$ is the saturation pressure,$\;R_{0}$ is the initial bubble radius, $R_{g}$ is the gas constant, and δ is the concentration boundary thickness.

Bubble nucleation happens because the flowing pressure of molten polymer decreases from the sprue to the mold cavity during the filling process. In Moldex 3D, the cell nucleation rate is expressed through an exponential function of the concentration (mass conservation) of dissolved gas, as follows [85]:

(8) $$J\left(t\right)=f_{0}{\left(\frac{2\gamma }{\pi M_{W}/N_{A}}\right)}^{1/2}exp\left[-\frac{16\pi \gamma^{3}F}{3k_{B}T\left(\frac{\bar{c}\left(t\right)}{k_{H}}-P_{C}{\left(t\right)}^{2}\right)}\right]N_{A}\bar{c}\left(t\right)$$

where $f_{0}$ and *F* are fitting parameters of the bubble nucleation rate equation, $\bar{c}$ is the average dissolved gas concentration, $N_{A}$ is the Avogadro number, $k_{B}$ is the Boltzmann constant, and $M_{W}$ is the gas molecular weight.

The average concentration of SCF dissolved in the polymer at a time t is given by following equation [85]:

(9) $$\bar{c}\left(t\right)V_{L0}=c_{0}V_{L0}-\int_{0}^{t}\frac{4\pi }{3}R^{3}\left(t-t^{\prime },t^{\prime }\right)\frac{P_{D}\left(t-t^{\prime },t^{\prime }\right)}{R_{g}T}J\left(t^{\prime }\right)V_{L0}dt^{\prime }$$

where $V_{L0}$ is the volume of the polymer matrix.

The viscosity of the polymer melt will be influenced by the SCF is dissolved in the polymer melt. Moldex 3D applies the modified Cross model with Arrhenius temperature dependence to describe the viscosity [88]:

(10) $${ɳ}_{p}\left(T,\dot{\gamma }\right)=\frac{{ɳ}_{0}\left(T\right)}{1+{\left({ɳ}_{0}\dot{\gamma }/\tau^{*}\right)}^{1-n}}$$

with

(11) $${ɳ}_{0}\left(T\right)=Bexp\left(\frac{T_{b}}{T}\right)$$

where *n* is the power law index, ${ɳ}_{0}$ is the zero shear viscosity, $\tau^{*}$ is a parameter that describes the transition region between zero shear rate and the power law region of the viscosity curve, and B is the pre-exponential factor.

Contrary to Moldex 3D, Moldflow uses the fitted classical nucleation model to describe the nucleation rate as follows [89]:

(12) $$J=F_{1}N{\left(\frac{2\sigma }{\pi m}\right)}^{1/2}exp\left[\frac{-16F_{2}\pi \sigma^{3}}{3kT{\left(P_{v}-P_{l}\right)}^{2}}\right]$$

where J is the nucleation rate, N is the Avogadro’s number, m is the molecular mass of the gas molecule, σ is the surface tension, k is the Boltzmann’s constant, T is the temperature, $P_{v}$ is the gas pressure in the polymer prior to bubble nucleation, $P_{l}$ is the polymer pressure, $F_{1}$ is a fitting parameter (correction factor of the Zeldovich factor), and $F_{2}$ is a correction factor of the free energy barrier of bubble nucleation.

The viscosity model of the polymer melt mixed with SCF in Moldflow is given by [90]:

(13) $$ɳ={ɳ}_{r}{\left(1-\emptyset \right)}^{v_{1}}exp\left(v_{2}c+v_{3}c^{2}\right)$$

where ${ɳ}_{r}$ is the viscosity of the polymer (without gas), ∅ is the volume fraction of the nucleated gas bubble, c is the initial gas concentration, and $v_{1}$, $v_{2}$, $v_{3}$ are data-fitted coefficients.

**Figure 13:**
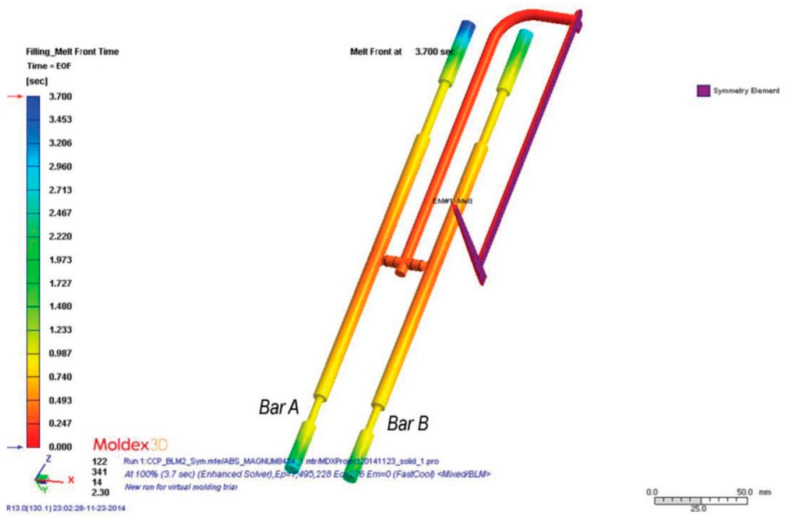
Melt front time in cylindrical bars simulated with Moldex 3D [80].

Modflow and Moldex 3D have been used to predict the morphology of the foam parts, injection cycle time, and to identify a set of optimal injection moulding parameters for a specific material. Gómez et al. [80] simulated the MuCell^®^ process of cylindrical bars (Figure 13) with Moldex 3D software and compared the numerical results with experimental ones. Similar results were obtained. Numerically (Figure 13), it was also possible to observe that the melt front time of bar A was longer than that of bar B, which means that bar B solidifies faster than bar A with less material. The authors also used Moldex 3D to simulate cell morphologies as well as the mechanical and fracture performance of microcellular PP/GF composites, and the results were very close to the experimental ones [74].

**Figure 14:**
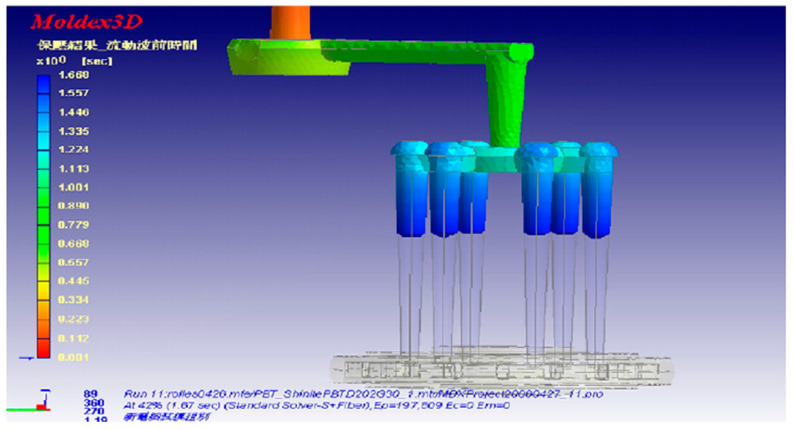
Runner system designed by Moldex 3D [91].

Hwang et al. [91] developed an optimal model for balanced flow characteristics, as shown in Figure 14. The sprue runner system of the mould used in experiments made the distance from each gate to the runner not constant, so there is an unbalanced melt flow problem, which resulted in rim thickness differences. Then, an optimal sprue and runner system in which the runner was in the middle of the cavity was designed. As observed, the samples moulded by this runner system have more balanced rim thickness compared to experimental parts.

As previously described, Moldex 3D and Moldflow, the most commonly used software to simulate injection moulding and related technologies, assume different models to describe the nucleation and the bubble growth behavior, and the viscosity models used to describe the behavior of the polymer melt mixed with SCF are also different. Therefore, we can expect different results from these two software tools. According to the literature, Moldex 3D seems to provide results closer to the experimental ones [74,80,84,92] and detailed simulation differences between two software were reported [93]. However, most simulation studies focused on relatively simple parts. On the other side, for the same part and moulding conditions, the simulation time of Moldflow can be much shorter than that of Moldex 3D, as Moldflow allows to import STL files, thus avoiding the need to generate the finite elements mesh for simulations [93]. Moreover, both software tools assume cell nucleation to be uniform and the SCF uniformly dispersed in the polymer melt, and consequently, cells are uniformly distributed across the part. However, in the filling stage of the MuCell process, the polymer melt mixed with SCF co-exists in different phases (single mixture, polymer/SCF, and mixed material containing bubbles), and this cannot be reproduced in the software. 

## 6. Conclusions and Prospects

MuCell^®^ is a relevant injection moulding technique to create light-weight plastic parts with a microcellular internal structure. This technique also allows producing parts with improved dimensional stability that enable reducing the injection pressure and clamping forces (energy savings) and the cycle time [16]. The produced parts exhibit lower shrinkage and warpage than conventional injected moulding parts [94]. Contrary to conventional injection moulding, where shrinkage is reduced by controlling both holding pressure and time, in the case of MuCell^®^, it is controlled by the SCF content and injection speed [94]. The main limitations are related to the surface quality and deterioration of mechanical properties. 

This injection moulding technique requires a proper control of different processing conditions (shot volume, mould temperature, gas dosage amount, and injection velocity) to reduce silver marks on the part surface and the production of plastic parts with different cell sizes distributed in different regions within the part inducing mechanical properties variations from region to region within the same part. Table 1 summarises the main effects of key processing conditions on cell morphology (e.g., size and density), skin thickness, weight reduction, and mechanical properties.

As discussed, there are strong links between the bubble nucleation and growth processes and the internal structure, surface quality, and mechanical properties. Aiming to improve the characteristics of microcellular injection moulded parts, different solutions have been proposed, either combining MuCell^®^ with other equipment or using different materials and additives. Improved surface quality and mechanical properties were obtained, but those solutions lead to complex mould structures and high costs and thus are not appropriated for mass production applications. 

As highlighted in this review, mould temperature and mould cavity pressure are the keys factors determining Mucell^®^’s part surface quality, determining both the solid skin layer thickness and foam zone characteristics (e.g., cell size and cell density), which regulate the apparent density of Mucell^®^ parts, weight reduction, and mechanical properties (e.g., tensile, impact, and flexural properties). Techniques such as gas counter pressure and dynamic mould temperature control have been used to improve the surface quality and to control the morphological structure of produced parts. The combined use of temperature and pressure sensors placed in the mould cavity to obtain relevant data for in-line process monitoring is also highly relevant [106]. Collected data can be used to determine in real time the rheological characteristics of the melt and, through the use of proper control systems and artificial intelligence tools, adjust processing parameters to optimise the injection process. However, in situ characterisation, also critical for real-time monitoring and process optimisation, is still a challenge. Tabatabaei et al. [107] used a mould with a transparent window and a high-speed digital camera to investigate cell nucleation and growth. However, the different thermal conductivity properties of glass and mould steel led to incorrect results. Recently, Zhao et al. [108] used an ultrasonic method for real-time analysis of cell size, surface roughness, and layer thickness. This technique was also used to measure clamping forces [109]. Together with artificial intelligence, the real-time data acquired by ultrasonic methods could open a new route to adjust on-time processing conditions, contributing to the development of a smart microcellular injection moulding approach. Nevertheless, better material databases and processing conditions–morphological development models are still required to allow the optimisation of microcellular injection moulding through the use of optimisation schemes based on the use of case-based reasoning, expert systems, fuzzy systems, Taguchi methods, genetic algorithm, or simulated annealing methods. 

Numerical simulation based on both Moldex 3D and Moldflow have been reported, aiming to improve the part properties, mould design, and process optimisation. However, better mathematical models capturing the complex mechanisms involved in the microcellular injection process are required. Currently, these simulation tools are not able to accurately simulate the entire injection process due to significant pressure variations, large cooling rates, complex flow fields, and complex nucleation mechanisms in the presence of fillers and additives. Cell nucleation is assumed to be uniform, and as a consequence, cells are uniformly distributed across the part. Therefore, better nucleation models are required. Moreover, it is not possible to obtain information on the cell shape, and this has an effect on the mechanical properties and anisotropy of the parts not captured by the software. Models that are able to consider the bubble convection mechanism, more accurate material data, and process condition models are also required. Due to current model limitations, current software tools are only able to predict with a certain level of accuracy cases where the material properties are well known, and the nucleation density can be considered uniform. Existing simulation tools are also not able to predict surface characteristics and mechanical properties.

The investment costs associated to MuCell^®^ represent a major limitation for the adoption of this technology. Strategies have been proposed based on the systems that do not require high-pressure pumps to bring CO_2_ and N_2_ to the supercritical state. Different approaches including the delivery of the gas from the gas cylinder to the molten polymer through an injector valve or the use of a high-pressure autoclave as a hopper [110]. These are cost-effective strategies but difficult to control and very efficient in terms of delivering the gas to the molten polymer. Recently, Trexel introduced a new tip-dosing module that eliminates the need for the special screw and barrel for foaming, allowing to reduce costs and to improve machine performance. 

Finally, the reduced clamp forces and injection pressures of MuCell^®^ in comparison to conventional injection moulding make it suitable to use additive manufacturing technologies to create inserts with conformal cooling channels, improving the performance of the injection moulds and part quality. Additive manufacturing has been explored as a rapid tooling strategy for several polymer processing technologies such conventional injection moulding, reaction injection moulding, and thermoforming, and the concept of hybrid moulds was fully discussed [111,112]. However, the use of additive manufacturing to produced advanced moulds for microcellular injection moulding has not been reported. 

## Figures and Tables

**Table 1:** The summary of the main effects of processing conditions on cell morphology, skin thickness, weight reduction, and mechanical properties (GF: glass fibre, PEI: polyetherimide, PPS: poly (phenylene sulfide), TPU: thermoplastic polyurethane, PA66: polyamide 66, PA6: polyamide-6, PS: polystyrene).

Material	Parameters	Changes	Morphology		Skin Thickness	Apparent Density	Weight Reduction	Warpage	Mechanical Properties									Reference
									Tensile			Impact	Flexural			Biaxial Bending		
			Cell Size	Cell Number (Density)					Elastic Modulus	Yield Strength	Tensile Strength		Young’s Modulus	Flexural Strength	Bending Stiffness	Maximum Force	Energy	
ABS	shot volume	↓	↓	↑	↓	↓	↑		↓	↓								[34]
	SCF content	↑	↓	↑	↓				↓	↓								
	mould temperature	↑	↓	↑	↑	no			no		no							
	injection velocity	↑	↓	↑	↓	no			no		no							
PP/GF	mould temperature	↑			↓				no				no	no	no	↓	↓	[73,74]
	degree of foaming	↓											↑	↑	↓	↓	↓	
	injection speed								not clear									
	delay time	↓			↓								↓	↓	↓	↑	↑	
	gas content	↓	↑						no									
	MuCell process pressure (MPP)																	
	shot volume	↓			↓	↓						↓	↓	↓				
PP, PP/CaCo3, ABS	SCF content	↑				↓							↓	↓				[95]
PEI	shot size	↑	↓	↑		↑	↓				↑	↑		↑				[96]
	SCF content	↑	↑	↓		↓	↑				↓	↓		↓				
	injection speed	↑	↓	↑		↑	↓				↑	↑		↑				
	mould temperature	↑			↓													
PPS/GF	injection speed	↑	no	no					↓		no	no						[97]
PPS	shot size	↑	↓	↑		↑					↑	↑	↑	↑				[98]
	SCF content	↑	↓	↑		no					no			no				
TPU	plasticising temperature	↑	↑		↑ until 200 °C then ↓				↑ until 198 °C then ↓									[99]
	injection speed	↑		↓	↑ until 45 ccm/s then ↓				↑ until 40 ccm/s then ↓									
	injection volume	↓																
	SCF content	↓	↑	↓	no													
HDPE/Wood fibre	gas content	↑		↑														[100]
	injection speed	↑		↑														
	mould temperature			↑														
	weight reduction	↓		↑					↑	↑								
PC	melt temperature	↑									↑	↑						[101,102]
	mould temperature	↑									↑	↓						
	MPP	↑									no	↓						
	SCF content	↑									↑	not clear						
	injection rate	↑									↑	not clear						
	shot size	↑									↑	not clear						
PA66/GF	injection temperature	↑	↓	↑														[103]
	gas injection pressure	↑				↓	↑						↑					
PA6	shot size	↑	↓	↓							↑							[104]
	melt temperature																	
	SCF content																	
	injection speed																	
PA6/Clay	shot size	↑	↑ until 18.4 mm then ↓	↑ until 18.4 mm then ↓							↑							[104]
	melt temperature	↑		no							no							
	SCF content	↑		no							no							
	injection speed	↑		no							no							
PP/GF	SCF content	↑				↓		↓										[105]
PP/talc	SCF content	↑						↓										[94]
PS	mould temperature	↑	↑	no	↓						↓	↓						[59,62]

## Data Availability

Not applicable.
